# Rheumatologists in Ecuador: Results of a Survey

**DOI:** 10.1155/2020/3421753

**Published:** 2020-12-19

**Authors:** Genessis Maldonado, Maria Intriago, Roberto Guerrero, Carlos Rios

**Affiliations:** ^1^MacNeal Hospital, Internal Medicine Department, 3249 S Oak Park Ave, Berwyn, IL 60402, USA; ^2^Universidad Espiritu Santo, 2.5 Km La Puntilla, Samborondón, Ecuador

## Abstract

**Introduction:**

Currently, there are no records of the current status of rheumatologists in Ecuador.

**Objective:**

The purpose of this study is to get to know the current status of rheumatologists in Ecuador, focusing on education, working conditions, productivity, distribution of time between work activities, and job satisfaction.

**Materials and Methods:**

A digital survey was created using the Google Forms platform. It was distributed to all rheumatologist members of the Ecuadorian Society of Rheumatology. The data analysis was carried out using the statistical program SPSS v.23®.

**Results:**

A total of 64 surveys were received. The response rate was 86.48%. 62.5% were men and 37.5% women, with an average age of 40.76 ± 9.18. The main workplace was state/public hospital (56.3%). The average working hours per week were 40.35 ± 25.72. Most rheumatologists in Ecuador (62.5%) received their training abroad. 79.7% of rheumatologists earn less than $ 49,000 annually. The mean retirement age was 66.51 ± 6.7, and 54.7% have a retirement plan. The average satisfaction of Ecuadorian rheumatologists was 5.4 ± 1.33 [0–7]; 17.2% are very dissatisfied with their annual income.

**Conclusions:**

This is the first recorded data on the characteristics of rheumatologists in Ecuador. Most rheumatologists obtained their specialist degree abroad. In general, rheumatologists in Ecuador are satisfied with their clinical practice and dissatisfied with their annual income and job security.

## 1. Introduction

Rheumatology was recognized as a medical specialty in the 20^th^ century; doctors Bernard Comroe and Joseph Lee Hollander were the first to introduce the term “Rheumatologist” in 1940 [1]. In Ecuador, the Rheumatology Society (SER) was created in 1960, which is one of the oldest scientific societies in the country [2]. Currently, SER has 74 specialists and in recent years, there has been an increase in the participation of young rheumatologists [3]. Approximately 90% of all rheumatologists belong to the Ecuadorian Society of Rheumatology [3].

The latest census data obtained in 2010 by the National Institute of Statistics and Censuses (INEC) report that Ecuador has 16.62 million inhabitants [4], and according to data from the Ministry of Public Health, there are approximately 29,734 general practitioners, which represents a rate of 18 doctors for every 10,000 inhabitants [5]. Furthermore, national figures for 2011 report around 19,083 medical specialists [5].

Currently, there are research articles on the well-being of rheumatologists in the United States [6]; in general, rheumatologists reported being satisfied with their practice as specialists, and it was shown that there is an increase of 1.2% in rheumatologists [7]. Maldonado et al. recently reported the current status of rheumatologists in Latin America and showed that rheumatologists in the region are very satisfied with their clinical practice and workplace, yet they are dissatisfied with their annual income and job security [7].

Dejaco et al. compared public health models from various countries in the region and determined that 1–3 rheumatologists per 100,000 inhabitants are needed. In Ecuador, we cannot determine this figure because we do not have the total number of rheumatologists; however, 74 rheumatologists are part of the Ecuadorian Society of Rheumatology.

Currently, we do not have information about the current status of rheumatologists in Ecuador. The purpose of this study is to get to know the current status of rheumatologists in Ecuador, focusing on education, working conditions, productivity, time distribution between work activities, and job satisfaction.

## 2. Materials and Methods

A digital survey was created using the Google Forms platform. It was distributed to all rheumatologist members of the Ecuadorian Society of Rheumatology. The data analysis was carried out using the statistical program SPSS v.23®.

The survey consisted of 45 questions that covered demographic data, comorbidities, clinical practice information, job satisfaction, and access to diagnostic techniques. Demographic variables included age, sex, marital status, number of children, race, country of birth, country of medical school graduation, and country in which their rheumatology fellowship was completed. The survey also asked about common comorbidities such as high blood pressure, diabetes mellitus, gastrointestinal, thyroid, autoimmune, and vascular diseases, gout, depression, and osteoarthritis.

Regarding clinical practice, the activities carried out in a work week, the number of weekly working hours, and the average annual income for the year 2017 were included. The survey also included questions about early access to an arthritis clinic, an infusion unit, X-rays, bone densitometry, and magnetic resonance imaging. Additionally, the survey asked if the participants had training in bone densitometry reading, ultrasound interpretation, and magnetic resonance imaging.

For job satisfaction, a visual analog scale graduated from 0 to 7 [8] was used. Six aspects of professional satisfaction were studied: practice satisfaction, job growth satisfaction, workplace satisfaction, annual income satisfaction, job security, and satisfaction with colleagues. The survey also asked about malpractice insurance, personal health insurance, and retirement plan.

### 2.1. Data Analysis

Duplicate responses were excluded, and the response rate obtained from the country was calculated using information provided by the Ecuadorian Society of Rheumatology secretariat regarding the number of rheumatologists as of October 2018.

The data were analyzed using the SPSS v23 statistical program. Frequencies, percentages, means, and standard deviation were calculated. The ANOVA test was used to analyze the differences between means, while the chi-square test was used for the categorical variables. Statistical significance was less than 0.05.

## 3. Results

Sixty four surveys of rheumatologist members of the Ecuadorian Society of Rheumatology (SER) were received. The response rate was 86.48%, and the demographic characteristics are described in Table 1. 70.3% (45) are engaged in adult rheumatology, 6.3% (4) are engaged in pediatric rheumatology, and 23.4% (15) practice internal medicine.

**Figure 1:**
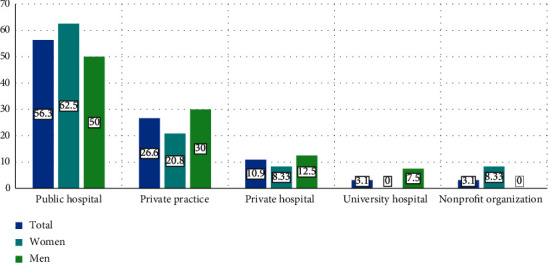
Main workplace of rheumatologists in Ecuador.

The main workplace was state/public hospital (56.3%) (36), followed by private practice (26.6%) (17), private hospital/clinic (10.9%) (9), university hospital (3.1%) (2), and nonprofit organization (3.1%) (2) (Figure 1). The average working hours per week were 40.35 ± 25.72. No significant differences were found in the workplace according to sex (*p* = 0.201).

Those who worked in a private hospital/clinic had a higher amount of working hours (46.12 ± 10.60) than rheumatologists in other workplaces such as state/public hospital (39.91 ± 21.60), private practice (34.17 ± 16.47), nonprofit organization (33.00 ± 10.30), and university hospitals (26.00 ± 9.79) (*p* = 0.005).

Regarding the type of patients they serve, 31.3% (20) of the patients had private insurance, 26.6% (17)had government insurance, 17.2% (10)had labor insurance, 4.7% (3) had another type of insurance, and 20.3% (11) did not have medical insurance.

In relation to other activities, 14% were dedicated to administrative work, 36% did teaching, 14.1% did clinical research, and 3.1% did clinical trials. It was found in women that 4.2% did administrative work (*p* = 0.078), 37.5% taught classes (*p* = 0.840), and 12.5% did clinical research (*p* = 0.781). Of the men, 20% did administrative work, 35% did teaching, 15% did clinical research, and 5% did clinical trials. There were no significant differences between men and women in relation to the activities performed.

84.4% (54) do not have access to an early arthritis clinic, and 51.6% (33) have access to an infusion unit. Regarding access to diagnostic tools, 82.8% (53) have access to X-rays, 43.8% (28) have access to a densitometer, 46.9% (30) have access to ultrasound, and 35.9% (23) have access to magnetic resonance imaging. However, most rheumatologists work in collaboration with other medical centers.

**Figure 2:**
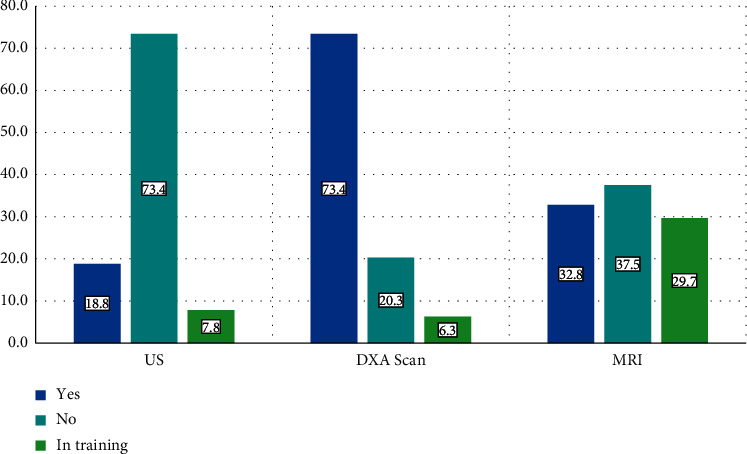
Training in the use of diagnostic tools.

Regarding training in the use of these diagnostic tools, 18.8% of rheumatologists have training in ultrasound, 73.4% in densitometric reading, and 32.8% in magnetic resonance imaging. Finally, 7.8% of rheumatologists are currently undergoing training in ultrasound, 6.3% in densitometric reading, and 29.7% in magnetic resonance imaging (Figure 2).

**Figure 3:**
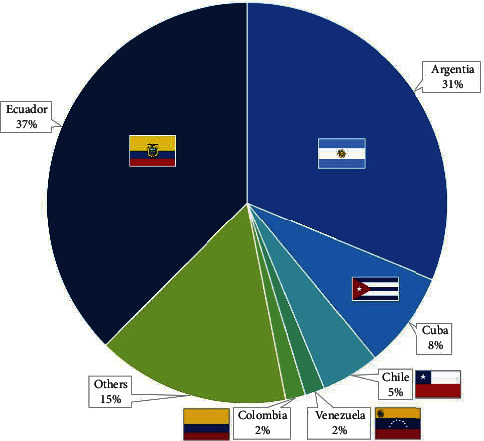
Countries in which rheumatology fellowship was obtained.

In regard to medical school education, the majority graduated as medical doctors in Ecuador (95.3%) (61). Regarding their postgraduate degree in rheumatology, 37.5% did their training in Ecuador and 62.5% abroad, the majority in Argentina (20) as seen in Figure 3.

In relation to income, 26.6% have an annual income of less than $ 19,000; 23.4%, $ 20–29,000; 17.2%, $ 30–39,000; 12%, $ 40–49000; 7.8%, $ 50–99,000; 9.4%, $ 100–149,000; 1.6%, $ 150–199,000; and 1.6%, $ 300–349,000.

**Figure 4:**
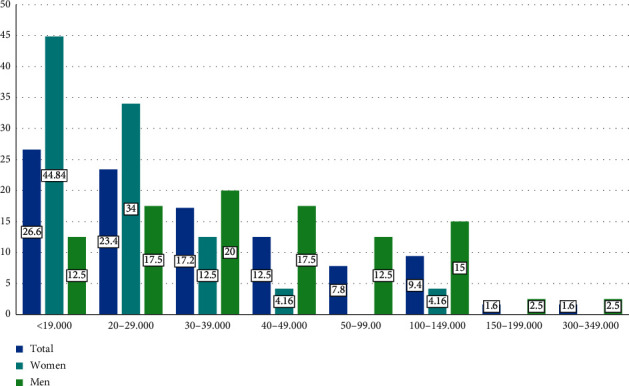
Annual income.

79.7% of rheumatologists earn less than $ 49,000 annually, while only 12.6% earn more than $ 100,000. Men have more income than women; 70% of men have an annual income above $ 39,000, compared to women where only 20.82% have an income of over $ 39,000 (Figure 4).

Regarding comorbidities, 24% had at least one. Among the most common comorbidities in men, osteoarthritis was observed in 81%, thyroid disease in 75%, and hypertension in 69%; while in the group of women, thyroid disease and osteoarthritis were evident in 29%, 14% had autoimmune diseases, and 7% had depression.

32.8% (21) have malpractice insurance, and 87.5% (56) have medical insurance. The mean retirement age was 66.51 ± 6.7, and 54.7% (35) have a retirement plan.

**Figure 5:**
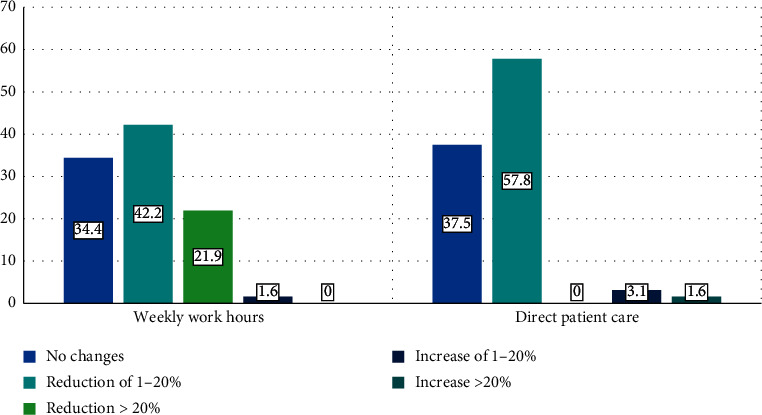
Desired changes in the clinical practice of Ecuadorian rheumatologists.

42.2% (27) of rheumatologists want a reduction in their working hours by 20%, and 57.8% (37) want a reduction in hours of direct patient care. 34.4% do not want to change their working hours, and 37.5% (24) do not want changes in patient care hours. 3.2% (2) would like to have an increase in working hours and direct care with patients (Figure 5).

The average satisfaction of Ecuadorian rheumatologists was 5.4 ± 1.33 [0–7]; 23.4% are very satisfied with their professional growth and 31.3% with their practice location; however, 17.2% are very dissatisfied with the annual income, 15.6% with job security, and 15.6% with their colleagues.

The degree of satisfaction was significantly higher in men in terms of general satisfaction (5.7 ± 1.1 vs. 4.9 ± 1.5, *p* = 0.018), professional growth (4.8 ± 2.0 vs. 3.7 ± 2.2, *p* = 0.041), location of practice (5.2 ± 1.9 vs. 3.8 ± 2.3, *p* = 0.013), annual income (4.5 ± 1.7 vs. 3.0 ± 1.8, *p* = 0.001), job security (4.5 ± 1.9 vs. 3.3 ± 2.1, *p* = 0.019), and with colleagues (4.8 ± 1.7 vs. 3.5 ± 2.3, *p* = 0.014).

## 4. Discussion

The demographic characteristics of rheumatologists in Ecuador show that the majority are mestizos, similar to what was reported in the population census of the National Institute of Statistics and Censuses, which showed that 71.9% of the Ecuadorian population are mestizos [12].

In the present study, it was evident that the majority of Ecuadorian rheumatologists who work in public hospitals and private practices have access to infusion units and X-rays. However, they do not have direct access to an early arthritis clinic, bone densitometry, magnetic resonance imaging, or ultrasound.

A similar study by Hogan and Bouchery [6] showed that 32% of US rheumatologists work in a private practice and 74% had access to infusion units, 64% to densitometry, 55% to X-rays, and 21% to an early arthritis clinic. These data are superior to the data presented in our study.

Likewise, in Canada, a study of rheumatologist found that 55% were dedicated to private practice and 32.6% in association with other physicians. The daily hours are mostly 9-10 working hours, and only 23.9% work 7 to 8 working hours a day [7], data similar to our average of 40 working hours per week. The Medscape Rheumatologist Compensation Report 2018 [8] described that 78% of rheumatologists dedicated 30–45 hours a week to see patients, while 96% dedicated more than 5 hours a week to administrative activities.

Regarding academic training, most of them studied medicine in Ecuador, while more than half completed their rheumatology fellowship abroad, mainly in Argentina. According to a study by the Pan American Health Organization (PAHO) [10], within the countries of Latin South America, there are rheumatology specialty programs in Cuba, the Dominican Republic, Costa Rica, Paraguay, Uruguay, Argentina, Bolivia, Brazil, Chile, Colombia, and Peru. Currently, Ecuador does not offer a rheumatology fellowship training.

Migration in Ecuador is an evident phenomenon, and it is estimated that 7.3% of the country's households have been affected by migration in search of work or study, especially to the United States, where it is estimated that around 2.5 million Ecuadorians live, and Spain where about half a million live [13]. The migration of health personnel is also a serious problem due to the leakage of investigative capacity and the deficit of personnel that they create in the health system [13]. A study found that of the doctors graduated in 2001 in Ecuador, 13.4% have left the country in search of specialization, the majority being men and from private universities [13].

Most rheumatologists in Ecuador receive an annual income of less than $ 30,000, which is significantly less than that found in other studies. According to data from the Ecuadorian Ministry of Public Health, an Ecuadorian doctor receives $ 1,676 to$ 2,967 per month, which is equivalent to an average of $ 28,000 per year [11]. In Hogan and Bouchery's study [3], only 9% earned less than $ 100K a year while in the Medscape Rheumatologist Compensation Report 2018, the annual income of rheumatologists in the United States was $ 259K [14]. A study about doctors' compensation in different countries showed that the United States is the country with the best annual remuneration with $ 313K, well above Spain with $ 63K, Brazil with $ 58K, and Mexico with $ 22K, showing that there is a large gap between the United States and Latin America [11]. From the region, it is estimated that Brazil and Chile have the highest salaries and Ecuador has an average salary between $ 10 and 26K, while Cuba and Venezuela have the lowest salaries.

## 5. Conclusion

This is the first reported data of rheumatologists in Ecuador. 63% completed their rheumatology fellowship abroad. In general, they are satisfied with their clinical practice, but they are dissatisfied with their annual income and job security. We consider that this study will enhance the interest of rheumatology in Ecuadorian young medical doctors.

## Figures and Tables

**Table 1:** Demographic characteristics.

Parameters	*n* (%)	*p* < 0.05
Female	24 (37.5)	0.003
Male	40 (62.5%)	
Age	40.76 ± 9.18	0.003

Race		
Mixed (mestizos)	58 (90.6)	0.297
White	4 (6.3)	
African American	1 (1.6)	
Indigenous	1 (1.6)	

Marital status		
Married	48 (75)	0.01
Single	11 (17.2)	
Living together	4 (6.3)	
Divorced	1 (1.6)	
Number of children	2 (0–5)	0.000

## Data Availability

Data supporting this research article are available from the corresponding author upon request.
